# Changes in DNA Methylation in Mouse Lungs after a Single Intra-Tracheal Administration of Nanomaterials

**DOI:** 10.1371/journal.pone.0169886

**Published:** 2017-01-12

**Authors:** Ali M. Tabish, Katrien Poels, Hyang-Min Byun, Katrien luyts, Andrea A. Baccarelli, Johan Martens, Stef Kerkhofs, Sven Seys, Peter Hoet, Lode Godderis

**Affiliations:** 1 Centre for Environment and Health, KU Leuven, Leuven, Belgium; 2 Integrated Cardio Metabolic Centre, Huddinge, Sweden; 3 Laboratory of Environmental Epigenetics, Exposure Epidemiology and Risk Program, Harvard School of Public Health, Boston, Massachusetts, United States of America; 4 Centrum voor Oppervlaktechemie en Katalyse, KU Leuven, Leuven, Belgium; 5 Laboratory of Clinical Immunology, KU Leuven, Belgium; 6 IDEWE, External Service for Prevention and Protection at work, Heverlee, Belgium; Suzhou University, CHINA

## Abstract

**Aims:**

This study aimed to investigate the effects of nanomaterial (NM) exposure on DNA methylation.

**Methods and Results:**

Intra-tracheal administration of NM: gold nanoparticles (AuNPs) of 5-, 60- and 250-nm diameter; single-walled carbon nanotubes (SWCNTs) and multi-walled carbon nanotubes (MWCNTs) at high dose of 2.5 mg/kg and low dose of 0.25 mg/kg for 48 h to BALB/c mice. Study showed deregulations in immune pathways in NM-induced toxicity *in vivo*. NM administration had the following DNA methylation effects: AuNP 60 nm induced CpG hypermethylation in *Atm*, *Cdk* and *Gsr* genes and hypomethylation in *Gpx*; *Gsr* and *Trp53* showed changes in methylation between low- and high-dose AuNP, 60 and 250 nm respectively, and AuNP had size effects on methylation for *Trp53*.

**Conclusion:**

Epigenetics may be implicated in NM-induced disease pathways.

## Introduction

Humans are exposed to airborne ultra-fine particles from different sources [1]. Human exposure to environmental stressors has changed because of the anthropogenic factors and more recently with rapid developments in nanotechnology, which is engineering nanomaterial (NM) with size-dependent properties called nanoparticles (NPs). NM possesses at least one dimension < 100 nm, whereas in NPs, all dimensions are < 100 nm [2]. NM, including gold NPs (AuNPs), titanium dioxide NPs, zinc oxide NPs, single-walled carbon nanotubes (SWCNTs) and multi-walled carbon nanotubes (MWCTs), are used in many applications. However, the widespread presence of these materials, small size and unique physicochemical properties also pose public health concerns [3]. The effects of NM in lungs and blood cells need to be investigated. Also, AuNPs and CNTs are introduced into the body in targeted drug-delivery systems, which raises questions about the fate and effects of this NM in the body.

Gold (Au) is considered relatively inert and biocompatible; however, recent studies raised concerns about the biocompatibility of Au in the nano-size range. AuNPs could have adverse effects by interacting with and damaging the vital cell components such as cell membrane, mitochondria and nucleus. In contrast, studies also reported AuNPs as non-toxic, so the results for AuNP-induced toxicity are contradictory [4]. The physicochemical properties of CNTs could pose health concerns similar to that observed with asbestos, such as the development of mesothelioma. They are also considered potentially carcinogenic in that SWCNT inhalation was shown to induce mutation in the k-ras oncogene locus [5]. Different mechanisms for the observed toxicity of AuNPs and CNTs include the induction of oxidative stress, DNA damage, and immune deregulation [6]. However, the epigenetic effects of NM have not been studied. We need to understand epigenetic hemostasis in response to NM exposure.

Epigenetic modifications (i.e., DNA methylation, histone modification, microRNAs) alter gene activity without altering the DNA sequence. DNA methylation (5-methylcytosine: 5mC) is one of the most-studied epigenetic modifications [7] and occurs almost exclusively on cytosine followed by a guanine base (i.e., CpG dinucleotides). CpGs are preferentially distributed within gene promoter regions, where they regulate gene expression [8]. Several classes of environmental chemicals, including metals, particulate matter, and endocrine/reproductive disrupters, modify gene promoter methylation marks [9]. Xenobiotic exposure affects global DNA methylation (total number of methylated cytosines in the genome) and global DNA hydroxymethylation (5-hydroxymethylcytosine: 5hmC) (total number of hydroxymethylated cytosines in the genome) [10, 11]. 5hmC recently gained interest because it represents the DNA demethylation pathway [11].

The current study aimed to investigate whether NM exposure to animals could result in epigenetic changes, that is, in global 5mC, 5hmC and gene-specific methylation.

## Materials and Methods

### Gold NPs and CNTs

Citrate-coated colloidal AuNPs of 3 primary sizes (small: 5 nm; medium: 60 nm, and large: 250 nm) were obtained from BBInternational (Cardiff, UK). SWCNTs (Raw Soot) were purchased from National Institute of Standards and Technology (NIST) (SRM: standard reference materials; 2483) (Gaithersburg, MD, US). AuNPs were thoroughly characterized for their primary particle diameter, hydrodynamic diameter and zeta-potential in baxter water (B.Braun Medical Inc, Irvine, CA, US) and in 2% serum by dynamic light scattering (DLS). MWCNTs (NM-400) were obtained from European Commission, Joint Research Centre, Institute for Reference Materials and Measurements (Milan, Italy). CNTs were characterized for their size distribution in H_2_O by electron microscopy [12].

### Preparation of NM

Stock suspensions (2 mg/ml) of powder samples (SWCNTs and MWCNTs) were prepared in pure H_2_O with 2% mouse serum by ultra-sonication (PTS Technics, Huddinge, Sweden) for 16 min as described [13]. NP stock suspensions were used within 1 h of preparation. Working concentration of CNTs and AuNPs (high dose: 1 mg/ml; low dose: 100 μg/ml) were prepared before instillation in saline solution with 0.2% mouse serum in lipopolysaccharide-free vials.

### Animals

Male BALB/c mice (~20 g, 7 weeks old) were obtained from Harlan (Horst, The Netherlands) and housed in filter cages in a conventional animal house at controlled temperature (21 ± 1°C) and humidity (50 ± 10%) with 12-h dark/light cycles. Mice were fed lightly acidified water and pelleted food (Trouw Nutrition, Ghent, Belgium) *ad libitum*. All experimental procedures were approved by the local ethics committee (Katholieke Universiteit Leuven, Leuven, Belgium) (project number ML8557).

### Experimental design

Mice were divided into 2 experimental groups for treatment: 1) vehicle control (*n* = 8), and 2) exposed. The exposed animals were administered AuNPs (*n* = 5/group): 5 nm, low dose (0.25 mg/kg) and high dose (2.5 mg/kg), 60 nm, low dose (0.25 mg/kg) and high dose (2.5 mg/kg), and 250 nm, low dose (0.25 mg/kg) and high dose (2.5 mg/kg); CNTs (*n* = 5/group); single-walled CNTs (SWCNTs), low dose (0.25 mg/kg) and high dose (2.5 mg/kg) and multi-walled CNTs (MWCNTs) low dose (0.25 mg/kg) and high dose (2.5 mg/kg).

Mice were anesthetized with isoflurane (3–5%) (Abbott Laboratories, SA Abbott NV, Ottignies, Belgium) for 2 min. Each mouse received 50 μl working NP solution or decitabine (1 mg/kg prepared in saline with 0.2% mouse serum) or vehicle (saline with 0.2% serum) by single intra-tracheal instillation with 1-ml syringes (BD, Erembodegem, Belgium) followed by 200 μl air. Sham control mice were also anesthetized and instilled with 250 μl air. Mice were weighed before instillation and examined after instillation until fully recovered from the anesthesia or any adverse effects (e.g., anxiety). After instillation, mice were transferred to the animal facility for 48-h exposure, then mice were weighed and killed by overdose of pentobarbital (90 mg/kg *i*.*p*.). Blood was sampled from the retro-orbital plexus and placed in K_3_EDTA-coated vials and flash-frozen in liquid nitrogen. Mice lungs were perfused with saline solution to clear blood cells. Mouse lung lobes were dissected, transferred into sterile vials, weighed and flash-frozen. Samples were stored at -80°C. We applied NMs to animals at subcytoxic and subgenotoxic levels and for 48 h to mimic short in-vivo exposure and the approximate doubling time of some lung cells. We were investigating DNA methylation changes, which could take place with DNA methyltransferase activation or inactivation. Also since ten-eleven translocation (Tet) genes could induce active methylation changes, so we needed to observe methylation changes in a relatively short exposure time (~48 h) rather than the higher duplication time of some lung cells.

### Bronchoalveolar lavage and bronchoalveolar lavage fluid (BALF) processing

BALF was sampled from mice and cytospin slides were prepared before perfusion as described [14]. BALF cells were stained with trypan blue dye (Invitrogen, Belgium) and total cells were counted under a light microscope. BALF cells were fixed on slides and stained with hematoxylin and eosin for differential random counting. Images of BALF cells were taken with a Zeiss Axiovert 220M microscope equipped with Axiovision 4.8.2 imaging software and a 100x oil lens with a 10x ocular lens.

### Lung cytokine measurements by cytometry bead array

Cytokine concentration (inteleukin 1 [IL-1], IL-4, IL-5, IL-6, IL-17a, KC) in lung tissue was measured from the middle lobe of the right lung from each animal by using cytometry bead array kits (CAB, BD biosciences, Belgium) on an LSR Fortessa flow cytometry platform equipped with FCAP Array v3.0 software (BD Biosciences, Belgium). Briefly, lung tissue was homogenized in lysis buffer and protein concentration in lysates was measured. Cytokine concentrations were calculated on the basis of standard curve data by using FCAP Array software (BD Biosciences).

### DNA damage measurement by Comet assay

DNA damage induced by NM administration was investigated in the inferior lobe of the right lung from each animal by the Comet assay in accordance with the standard protocol “European Network on the Health and Environmental Impact of Nanomaterials” by the ENPRA project (risk assessment of engineered NPs). All experimental processing for the Comet assay was performed at 4°C in dark. Comet assay consumables were purchased from Trevigen Inc. (Gaithersburg, MD, US). BALF was centrifuged at 2000xg for 10 min. Cell pellets were resuspended in 250 μl saline solution, and 5 μl of this solution mixed with LM agarose was applied on the Comet slides. LM agarose was allowed to set for 10 min and slides were immersed in the cell lysis solution for 1 h. Then, slides were immersed in alkaline unwinding solution (30 min) before electrophoresis at alkaline condition (pH > 13) for 30 min at constant voltage. Slides were washed in H_2_O, dehydrated in 70% ethanol for 5 min, dried, stained with cyber green in TE buffer, and imaged by fluorescence microscopy (Olympus Corp., Tokyo). Images were analyzed by using Autocomet (TriTek Corp, Sumerduck, VA, US).

### Oxidative stress measurement by liquid chromatography-mass spectrometry (LC-MS)

The superior lobe of the right lung from each animal was minced while being immersed in 1 mM N-ethylmaleimide (NEM), a blocking thiol agent to prevent rapid oxidation of GSH. Reduced glutathione (GSH) and oxidized glutathione (glutathione disulfide, GSSG) were measured by an LC-MS in-house developed method, based on the method of Guan et al., 2003 [15] in part. The LC-MS analysis involved a Waters Acquity UPLC coupled to a Micromass MS Technologies Quattro Premier mass spectrometer with electron spray ionization (ESI). The LC separation involved a Waters Acquity UPLC BEH C18, 50 mm × 2.1 mm, 1.7 μm column, held at 40°C.

### Global DNA methylation/hydroxymethylation measurement by LC-MS

The left lung from each mouse underwent epigenetic analysis. 5-methylcytosine (5mC) and hydroxymethylation (5hmC) assays in mice lung DNA samples (DNA isolated by using Qiagen Blood and Tissue kits; Qiagen, Venlo, the Netherlands) were performed as described [16]. An amount of 1 μg DNA was hydrolyzed into individual nucleosides, and samples underwent LC-MS to quantify the absolute amount of 5mC and 5hmC in control and exposed lung DNA samples because not enough DNA was obtained from mouse blood samples for global 5mC and 5hmC analysis. 5mC is expressed as a percentage of 5-methylcytosine to total number of cytosines present in the genome. 5hmC is expressed as percentage of 5-hydroxymethylcytosine to total number of cytosines present in the genome.

### Gene-specific DNA methylation measurements by bisulfite pyrosequencing

For the analysis of gene-specific methylation, 17 genes were selected for DNA promoter methylation profiling after exposure to NM. Genes were selected; for example, *Gsr*, *Gpx* and *Gss* [17] that are involved in oxidative stress response pathway. Genes were also selected from immune pathway [18, 19], cell cycle regulation pathways [20] and DNA methylation pathways (S1 Table) [21, 22]. CpGs within the promoter region of the selected genes were targeted for methylation analysis (S2 Table). Bisulfite-PCR pyrosequencing assays (n = 17) were designed for the selected genes (S3 Table). Gene-specific methylation analysis was of DNA from the left lung and blood samples from each mouse. Genomic DNA was treated by using the EZ DNA methylation Gold kit (Zymo Research, Orange, CA, USA). Final elution volume was 40 μl with M-elution buffer. PCR was performed with 30 μl volume and 15 μl GoTaq Green Master mix (Promega), 10 pmol forward and 10 pmol reverse primers, 50 ng bisulfite-treated DNA, and water for a 30-μl final volume. Amplicons were analyzed in 2% agarose gel. Pyrosequencing was performed as described [23]. PCR and sequencing assays information are in S3 Table. We designed a control oligo for 100% DNA methylation (PSQ-C oligo), 0% DNA methylation (PSQ-T oligo) and the sequencing primer for the control oligo. We mixed PSQ-C oligo (or PSQ-T oligo) with the sequencing oligo in PyroMark Annealing Buffer (QIAGEN Inc., Valencia, CA) for pyrosequencing (S4 Table). The methylation level is expressed by % 5mC.

### Statistical analysis

Wilcoxon test with Dunn all-pairs post-hoc analysis with JMP 10 (SAS Inst., Cary, NC, USA) was used to compare exposed and control groups. To investigate the effect of NM exposure on gene-promoter methylation, we used a three-step statistical approach with pyrosequencing data. In the first step, we used average methylation values for all CpGs analyzed for a gene. Average methylation for all CpGs per gene as a dependent variable and exposure groups as independent variables was analyzed by Wilcoxon test and p-values were obtained. In the second step, we performed statistical analyses per CpG methylation. Each CpG methylation within a gene (e.g., Atm CpG#1) as the dependent variable and exposure groups as independent variables were analyzed by Wilcoxon test and p-values were obtained. In the third step, we used Dunn all-pair post-hoc analysis of significant methylation data in the previous steps to investigate exposure parameters (i.e., AuNP size and exposure dose, CNT shape and exposure dose) responsible for the observed variance in methylation in lung and blood DNA. P<0.05 was considered statistically significant. Analysis involved use of SPSS.v22 (SPSS Inc., Chicago, IL).

## Results and Discussion

### Results

#### NM characteristics

The physicochemical properties of NM we used were thoroughly characterized for their size and charge distribution (S5 and S6 Tables, S1 Fig). AuNPs showed negative zeta potential in H_2_O and 2% serum and a size-dependent increase in negative zeta potential in H_2_O but not serum.

#### Immunotoxicity of NM

**Fig 1:**
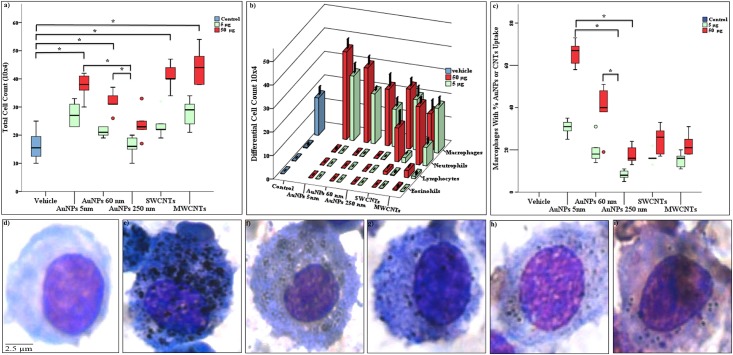
Bronchoalveolar lavage (BAL) fluid analysis in mice exposed to nanomaterial. a): total cell count; b): differential cell count.; c): uptake/association by/with BAL macrophages. For clarity of presentation in panel b, significant groups are not annotated. In panel b, macrophages count was significant in following exposure groups: AuNPs 5nm 50μg and AuNPs 60nm 50μg compared to the vehicle; AuNP 5nm 50μg compared to AuNP 250nm 5μg; and AuNPs 60 nm 50μg compared to the AuNP 250nm 5μg dose categories. Neutrophils count was significant in following exposure groups: SWCNT 50μg and MWCNTs 50μg compared to vehicle. Lymphocytes count was significant in following exposure groups: SWCNT 50μg and MWCNTs 50μg compared to vehicle. In panel c, 100 macrophages were randomly counted for the microscopic presence or absence of NM aggregated inside the cytoplasm at 1000X magnification. Representative images of macrophage d): vehicle; e): AuNPs 5nm; f): AuNPs 60nm; g): AuNPs 250 nm; h): SWCNTs; i): MWCNTs. In panel a and c; the box plot describes the median (line across the box), inter-quartile range and maximum and minimum values (whiskers). Outliers are shown as colored circles outside the ends of whiskers. Data in panel b is represented as median ±SD. Asterisk sign (*) shows significance levels at *p* = 0.05 (dunn’s statistics). Gold nanoparticles: AuNPs; single-walled- and multi-walled carbon nanotubes: SWCNTs and MWCNTs.

To examine the immunotoxicity of NM exposure, we examined total and differential changes in cell counts in BALF. Total cell count was higher with exposure to AuNPs and CNTs than no exposure (Fig 1a). Macrophage and neutrophils showed dominant influx into lung interstices with AuNP and CNT exposure as compared with controls (Fig 1b). In sham and vehicle control groups, no macrophages had taken up NPs; therefore, they are not visible in Fig 1c. Macrophages from exposed and control mice showed that AuNPs and CNTs were dose-dependently taken up or or associated with BALF cells (Fig 1d–1i). Levels of selected cytokines in mouse lungs did not differ with AuNP and CNT exposure as compared to controls (S2 Fig).

#### Oxidative stress and DNA damage effects

NP-exposed mice did not show induced oxidative stress or DNA damage as compared to control mice (S3 and S4 Figs).

#### Global DNA methylation and hydroxymethylation in mouse lungs

**Fig 2:**
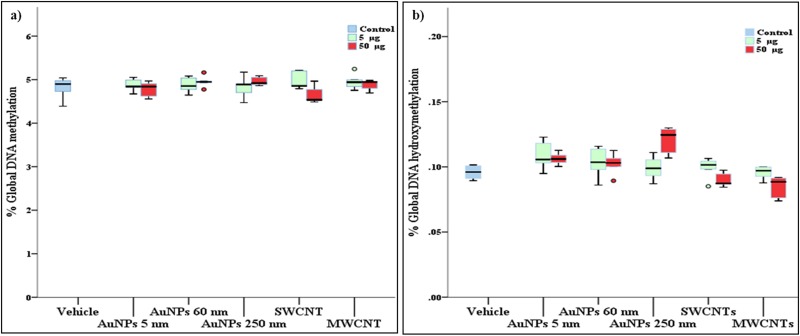
Global DNA methylation (5mC) and hydroxymethylation (5hmC) in lungs. a): no significant effects (Wilcoxon test) of gold nanoparticle (AuNPs) and single-walled and multi-walled carbon nanotubes (SWCNTs and MWCNTs) were observed on 5mC (*p* = 0.667 and 0.284 respectively). b): also no significant effect of AuNPs exposure on lung 5hmC were observed (*p* = 0.107). However, CNTs exposure showed significant effect on 5hmC (*p* = 0.024) levels by Wilcoxon statistics, while no group remained significant after multiple comparisons (Dunn all pairs post-hoc). In panel a and b; box plot describes the median (line across the box), inter-quartile range and maximum and minimum values (whiskers). Outliers are shown as colored circles outside the ends of whiskers.

AuNP and CNT exposure had no effect on on 5mC and 5hmC levels in mouse lungs (Fig 2a and 2b).

#### Gene-promoter methylation in mouse lungs and blood

The effect of NM exposure was investigated in mouse lungs exposed to AuNPs and CNTs (S7a and S7b Table). The effect of NM exposure on average gene promoter methylation and promoter CpG methylation was profiled in blood cells of mice exposed to AuNPs and CNTs (S8a and S8b Table).

**Fig 3:**
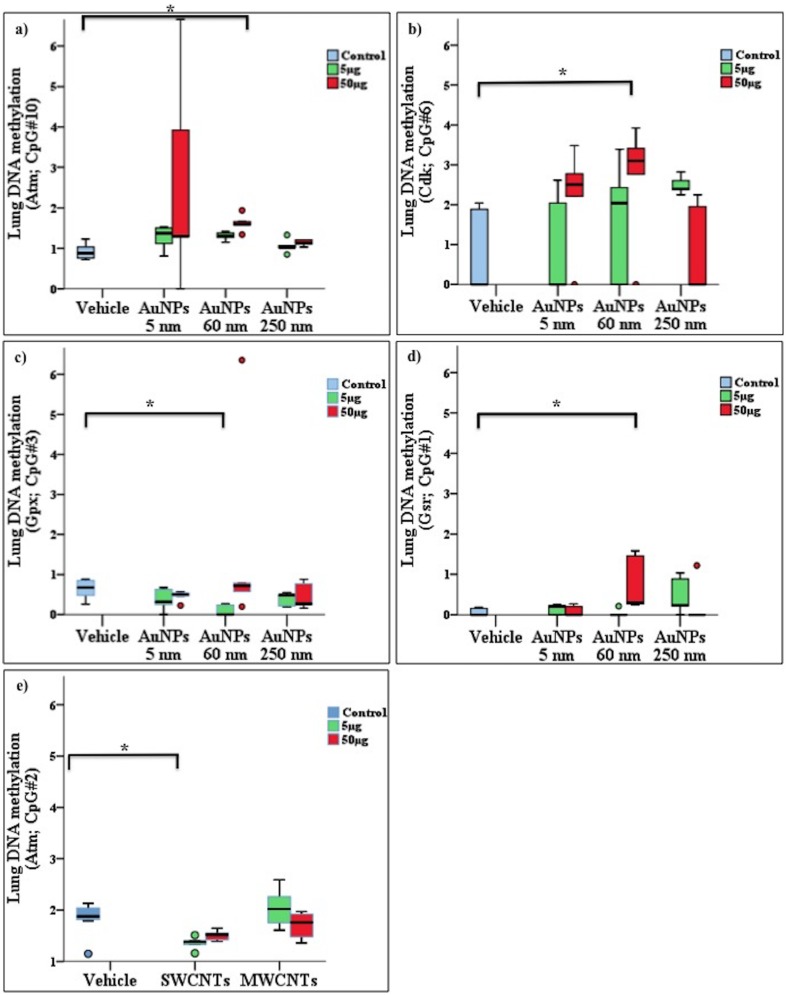
Effect of nanomaterial (NM) exposure on gene promoter methylation. Bars connect exposure groups with significant methylation difference, a-d): effects of gold nanoparticles (AuNPs) exposure on promoter methylation levels of *Atm* (a), *Cdk* (b), *Gp x*(c), and *Gsr* (d) genes in lungs. Effect of single and multi-walled carbon nanotubes (SWCNTs, MWCNTs) exposure on gene promoter methylation levels of *Atm* (e) gene in lungs. In panels, box plot describes the median (line across the box), inter-quartile range and maximum and minimum values (whiskers). Outliers are shown as colored circles outside the ends of whiskers. Asterisk sign (*) shows significance levels at *p* = 0.5 (dunn’s statistics). *Atm*: ataxia telangiectasia mutated; *Cdk*; cyclin-dependent kinase; *Gsr*: glutathione reductase; *Gpx*: glutathione peroxidase.

Dunn all-pair testing was performed to determine the effects of exposure parameters (i.e., NP size, CNT shape and dose) on promoter methylation. For exposure parameters with significant or borderline significant effects on average gene methylation and CpG methylation, we presented the results in Table 1. Also, Fig 3 shows the effect of NM exposure on gene promoter methylation.

**Table 1:** Effect of nanomaterial (NM) exposure variables on methylation of promoters of genes.

Exposure	Tissue, Gene symbol, (CpG)	Variable, DNA methylation, mean (95%CI), n	Variable, DNA methylation, mean (95%CI), n	*p-*value
Total exposure	Lung, *Atm*, (CpG#10)	Vehicle, 0.91 (0.76–1.06), n = 8	AuNP 60 nm 50 μg, 1.62 (1.36–1.89), n = 5	0.002
	Lung, *Cdk*, (CpG#6)	Vehicle, 0.72 (-0.11–1.55), n = 8	AuNP 60 nm 50 μg, 2.63 (0.73–4.54), n = 5	0.031
	Lung, *Gpx*, (CpG#3)	Vehicle, 0.64 (0.45–0.82), n = 8	AuNP 60 nm 5 μg, 0.10 (-0.07–0.27), n = 5	0.041
	Lung, *Gsr*, (CpG#1)	Vehicle, 0.06 (-0.01–0.13), n = 8	AuNP 60 nm 50 μg, 0.77 (-0.08–1.62), n = 5	0.034
	Lung, *Atm*, (CpG#2)	Vehicle, 1.84 (1.58–2.09), n = 8	SWCNTs 5 μg, 1.35 (1.19–1.51), n = 5	0.038
Effect of dose	Lung, *Gsr*, (CpG#4)	AuNP 60 nm 5 μg, 0.48 (-0.12–1.09), n = 5	AuNP 60 nm 50 μg, 1.76 (1.36–2.16), n = 5	0.018
	Lung, *Gsr*, (CpG#6)	AuNP 60 nm 5 μg, 0.04 (-0.07–1.15), n = 5	AuNP 60 nm 50 μg, 1.11 (0.51–1.71), n = 5	0.012
	Lung, *Trp53*, (CpG#1)	AuNP 250 nm 5 μg, 1.19 (0.45–1.93), n = 5	AuNP 250 nm 50 μg, 0.04 (-0.07–0.16), n = 5	0.028
	Blood, *Pparg*, (CpG#3)	AuNP 60 nm 5 μg, 1.18 (0.50–1.85), n = 5	AuNP 60 nm 50 μg, 4.81 (-0.95–10.57), n = 5	0.031
Effect of diameter	Lung, *Trp53*, (CpG#1)	AuNP 60 nm 5 μg 0.05 (-0.09–0.19), n = 5	AuNP 250 nm 5 μg, 1.19 (0.45–1.93), n = 5	0.034
Effect of shape	Lung, *Atm*, (average: CpG#1–6)	SWCNTs 5 μg, 1.18 (1.04–1.32), n = 5	MWCNTs 5 μg, 1.84 (1.46–2.22), n = 5	0.031

*p*-values computed by Mann-Whitney U statistics. AuNP: gold nanoparticles, CNT: carbon nanotubes, SWCNT: single-walled CNTs, MWCNT: multi-walled CNTs, n = number of mice.

* genomic position of CpGs are in S2 Table.

**Fig 4:**
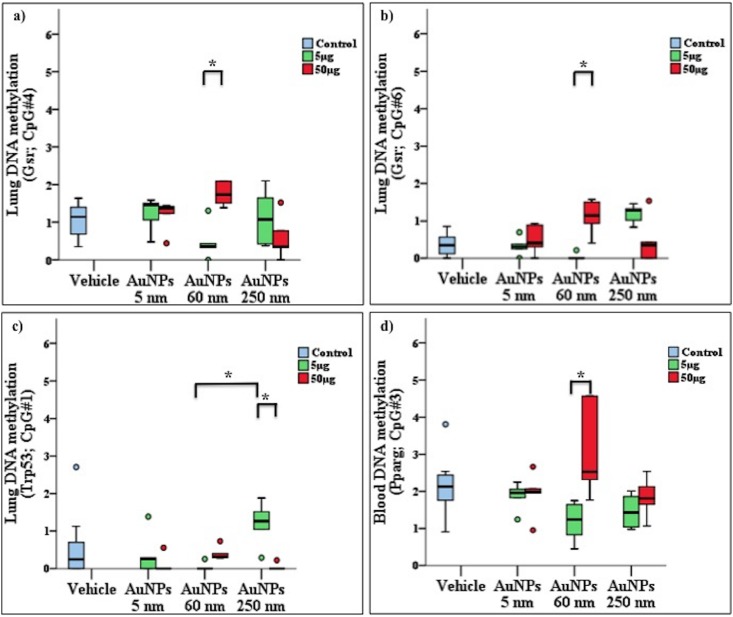
Effect of nanoparticles (NPs) dose and size on gene promoter methylation upon exposure. Bars connect exposure groups with significant methylation difference, a-d): effects of gold NPs (AuNPs) exposure dose on promoter methylation levels of *Gsr* (a, and b), *Trp53* (c) in lungs, and *Pparg* (d) genes in blood. AuNPs size effect on CpG methylation of *Trp53* gene was observed between 60 nm and 250 nm AuNPs. In panels, box plot describes the median (line across the box), inter-quartile range and maximum and minimum values (whiskers). Outliers are shown as colored circles outside the ends of whiskers. Asterisk sign (*) shows significance levels at *p* = 0.5 (dunn’s statistics). *Gsr*: glutathione reductase; *Trp53*: tumor protein P53; *Pparg*: peroxisome proliferator-activated receptor gamma.

**Fig 5:**
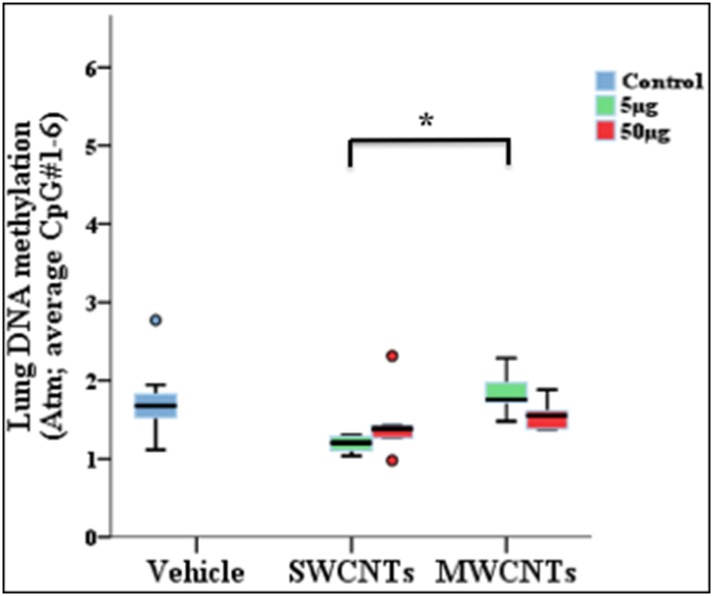
Effect of shapes of single and multiwalled carbon nanotubes (SWCNTs, MWCNTs) upon exposure on *Atm* gene methylation. Bars connect exposure groups with significant methylation difference. In panels, box plot describes the median (line across the box), inter-quartile ranges and maximum and minimum values (whiskers). Outliers are shown as colored circles outside the ends of whiskers. Asterisk sign (*) shows significance levels at *p* = 0.5 (dunn’s statistics). *Atm*: ataxia telangiectasia mutated; *Trp53*: tumor protein P53.

Compared to the vehicle group, AuNP 60 nm exposure in mouse lung tissue induced promoter hypermethylation in the genes ataxia telangiectasia mutated (*Atm*) (CpG#10, *p* = 0.002), cyclin-dependent kinase (*Cdk*) (CpG#6, *p* = 0.031) and glutathione reductase (*Gsr*) (CpG#1, p = 0.034) genes but promoter hypomethylation in glutathione peroxidase (*Gpx*) (CpG#3, *p* = 0.041) (see Fig 3, Table 1). As well, SWCNT exposure induced promoter hypomethylation in *Atm* (CpG#2, *p* = 0.038) (Fig 3e, Table 1). AuNP exposure had dose-specific changes in promoter methylation of *Gsr* (CpG#4 and CpG#6, *p* = 0.018 and *p* = 0.012, respectively), *Pparg* (CpG#3, *p* = 0.031) and tumor protein P53 (*trp53*) (CpG#1, *p* = 0.028) with low and high dose, 60 and 250 nm, respectively (i.e., dose effect) (Fig 4, Table 1). AuNP had a size effect on promoter methylation of *Trp53* (CpG#1, *p* = 0.034) (Fig 4c, Table 1). SWCNTs and MWCNTs had shape effects on promoter methylation of *Atm* (average: CpG#1–6, *p* = 0.031) (Fig 5, Table 1). AuNPs at 5 nm did not significantly affect CpG methylation as compared with controls. DNA methylation values (5 readings per group) had a greater spread for for *Atm*, CpG#10 (50 μg) and *Cdk*, CpG#6 (5 μg), so the extreme values were not computed as outliers. However, in general, all readings per animal including outliers (of respective groups) were included in the analysis.

## Discussion

Reports of nanotoxicology have cited several adverse effects associated with NM exposure (e.g., cytotoxic effects, immunologic effects and genotoxic effects) [24–26]. However, the epigenetic effects associated with NM exposure were not described. For insight into NM-induced cellular pathophysiology, we conducted cellular and epigenetic assays and found that NM exposure could alter the epigenetic status of genes involved in different cellular processes in mouse lung and blood cells.

NM administration altered BALF total cell count in exposed mice. Differential cell count revealed a macrophage-dominant immune response with AuNP administration and a macrophage- and neutrophil-driven immune response with CNT administration. These results suggest pulmonary inflammation in mouse lungs in response to NM exposure. Our findings agree with previous studies showing that NM exposure changed macrophage and neutrophil concentrations and engulfed the exposed NM after intra-tracheal instillation *in vivo* [27–29]. Macrophages invade lung interstices for cleaning function and take different phenotypes based on environmental signals, which might lead to pulmonary pathologies [30].

We did not observe changes in cytokine concentrations in lung interstices after NM exposure. However, studies have reported NM exposure in BALB/c mice leading to changes in cytokines concentrations in lung tissue [31]. One way to explain it, is through the time effect. Cytokines usually have a short life span (e.g., ~103 and ~70 min for IL-6 and tumor necrosis factor alpha, respectively, in humans) [32]. The life span is greater for inflammatory cells such as macrophages and neutrophils than cytokines. Inflammatory cells may be detected after 48 h of a single NM exposure, but cytokines are absent or diluted during this time window, which suggests that clearance occurs earlier for cytokines than inflammatory cells. We previously observed this mechanism, whereby NM exposure led to significant changes at cellular levels but not many significant changes in cytokine levels [33]. Furthermore, some studies also showed the contradictory effects of NM exposure on inflammatory endpoints under different experimental settings [4]. These observations highlight the need for standardized NM exposures applied under similar experimental settings to draw conclusions across studies.

Our investigations of oxidative stress showed that NM exposure at selected doses did not significantly alter GSSG/GSH ratio or DNA damage in BALF cells from exposed mice. Because of the use of different doses, exposure methods, *in vitro* and *in vivo* models, and even AuNPs and CNTs purchased or manufactured from different sources or by different methods, comparing our findings with other publications describing oxidative and genotoxic damage induced by NM is difficult [34], [35]. Here we used sub-cytotoxic CNT doses for a relatively short exposure time (48 h), which could explain in part the contradiction with published studies on the observed effects.

An important part of the current study was to investigate the epigenetic alterations induced by NM exposure. Gene-specific methylation was analyzed in mice lung and blood DNA by bisulfite-PCR pyrosequencing, and global 5mC and 5hmC was analyzed by LC-MS only in mouse lung DNA because of insufficient yield of DNA from blood. NM exposure had no significant effect on global lung 5mC and 5hmC levels. Our results suggest that BALB/c lungs are insensitive to global DNA methylation changes with NM exposure at the selected doses and time-point. NM has an aggregation tendency, and to be consistent and coherent, we always used tissue biopsies taken at the same time for each analysis (e.g., left lung always used only for DNA methylation analysis). One other reason for using different lung lobes for different experimental endpoints was that tissue processing for one analysis could pose risk in other analyses. For example, we crushed right lung lobes and processed them for cytokine analysis. However, this processing might affect the DNA methylation, which implies that similar tissue processing, and hence pooling of lung tissue, might not be optimal for the different analyses we performed.

For gene-specific methylation, AuNP size and CNT shape and dose affected gene-promoter methylation in exposed mice. In general, more genes were epigenetically altered in lungs than blood of exposed mice. This is expected, because exposure was direct for lung cells but not blood cells. Furthermore, more genes were epigenetically altered with AuNPs than CNTs (Table 1). This finding contrasts with the paradigm that exposure to AuNPs does not induce an adverse biological response [36, 37]. From our findings, AuNPs may be more potent than CNTs in inducing epigenetic changes. More genes were epigenetically affected by AuNP 60 nm than 5 or 250 nm. AuNPs used in this study were citrate-coated. AuNPs with 5-nm diameter have higher surface area for the attachment of citrate than those with 60- and 250-nm diameter. High contents of citrate on the surface of AuNPs with 5-nm diameter may mask the effects of these NPs on gene promoter methylation. These 5-nm AuNPs also have higher tendency to agglomerate in the biological media than AuNPs with 60- and 250-nm diameter (see S1 Fig), which could also lead to suppressed biological activity.

Comparing our findings with previous data is difficult, especially for gene-specific methylation endpoints. One study investigated the effect of nano-silicon dioxide (nano-SiO2) and found hypermethylation of poly [ADP-ribose] polymerase 1 (*PARP-1*) *in vitro* [38]. No other studies investigated the effect of NM exposure on gene methylation. We are the first to report that genes are sensitive to methylation changes by the nature, size, shape and dose of NM exposure *in vivo*. Our statistical analysis included the assessment of DNA methylation changes at CpG levels within the selected region of gene promoter sites. We found induced CpG-specific methylation changes in NM-exposed mice, which highlighted the differential sensitivity of individual CpGs within gene promoters to NM.

Previous reports described altered histone posttranscriptional modifications and microRNAs in response to NM exposure *in vitro* [39, 40] and we found altered DNA methylation in response to NM exposure. Thus, epigenetic machinery as whole (i.e., DNA methylation, histone posttranscriptional modifications and microRNAs) are susceptible to alterations on NM exposure. An interesting observation was changes in CpG methylation in *Trp53* (due to AuNP size) and *Gsr* (due to AuNP dose) genes where high dose and larger particles size lead to CpG hypermethylation. Both genes are important in stress response pathways. Products of *Gsr* are important in resisting oxidative stress and *Trp53* is important in regulating the cell cycle in response to stress. Though we did not profile gene expression in this study, *Gsr* and *Trp53* CpG hypermethylation could point that cells are stress sensitized via *Gsr* and *Trp53* methylation induced down-regulation of gene expression. This could implicate that DNA methylation deregulations could render cells epigenetically sensitive to NM.

In current study, DNA methylation changes were investigated at a single time-point of 48 hours after the nanomaterial administration, which is relatively modest exposure time in order for immune infiltrates to be observed in the lung interstices [41]. However, multiple time-points study could help in identifying sensitive time-windows where cells are more epigenetically responsive to nanomaterial. It could also help us to identify how nanomaterial interact with cells over longer time period i.e., it is possible that acute exposure of nanomaterial induces epigenetic changes via the production of reactive oxygen species (ROS), while chronic exposure of nanomaterial could interact with the mitotic machinery (aneugenic activity) of the cell, or could induce DNA lesions by chemically modifying/breaking the DNA molecules (clastogenic activity). However, in current setting we did not observe genotoxicity in lungs administered with nanomaterial, which implies these nanomaterials in the current settings may not have exerted epigenetic changes via genotoxic mechanisms. Alternatively, observed DNA methylation changes might be linked with increased production of ROS, however, we did not observe increased ROS production as measured by GSSG/GSH ratio (S3 Fig) in lung tissue. Other proposed mechanisms of observed epigenetic effects could be the interaction of nanomaterial with DNA methylation enzymes, which may alter activities upon xenobiotic exposure [42, 43]. A relatively easy system to study DNA methylation kinetics after nanomaterial exposure could be a cell free system i.e., nanomaterial exposure to fully methylated or fully unmethylated DNA molecule in the presence and/or absence of DNA methylation modifying enzymes, such as DNA methyltransferases and CpG-binding proteins. This system could allow us to quantify the direct impact of nanomaterial on DNA methylation as well as impact of nanomaterial on the activity of DNA methylation modifying enzymes at multiple time-points. On a functional level, CPG methylation changes could lead to expression changes and/or even changed splicing patterns of the affected genes. To understand the phenotypic relevance of epigenetic changes observed in NM-exposed cells, the gene expression activity may need to be measured along with epigenetic changes. However, in the current study, gene expression effects were not studied, which limits highlighting the functional relevance of the epigenetic changes we observed. DNA methylation-induced expression changes in response to NM administration need further investigation. Equally important is to delineate what epigenetic response could be considered toxic (e.g., a differential methylation signature at a single CpG locus) rather than an adaptive response. The epigenetic sensitivity of individual lung cell types to NM exposure would be of interest.

In the current study, we included 17 genes for promoter methylation analysis after administration of gold nanoparticle and carbon nanotubes to mice. These genes are commonly affected by xenobiotic exposure [44]. The selected NM exposure may induce promoter methylation changes in genes other than selected in the current study, and it is also further needs to investigated if other nanomaterials (e.g., zinc oxide nanoparticles, silver oxide nanoparticles, titanium based nanomaterials) could also lead to DNA methylation changes similar to what is observed in current study for gold nanoparticles and carbon nanotubes. Generalization of current findings to the broader population of nanomaterials requires further research. Also, Investigations are needed to understand the functional importance of these epigenetic changes in nanotoxicology. Recently, cellular epigenetic stress was proposed to drive the disease process in hepatocellular carcinoma [45]. Our findings agree in that we observed altered gene-promoter methylation in NM-exposed mice without genotoxic effects. However, the extent and duration of epigenetic stress leading to diseases needs to be fully understood.

## Conclusions

Our study revealed that NM exposure could lead to epigenetic changes in mouse lung and blood. These observations warrant further investigations of the NM effects on epigenetics. Also, further studies should include broader populations of NM and different time points and model organisms to comprehend how NM interacts with epigenetics. We revealed induced bronchial inflammation in response to NM exposure. The reverse causality between observed DNA methylation changes in lung DNA and bronchial inflammation needs further investigation.

## Supporting Information

S1 Fig Dynamic light scattering size distribution of gold nanoparticles (AuNPs) 5 nm (a), 60 nm (b) and 250 nm (c) in H_2_O (blue curve) and 2% serum (red curve). (TIF)

S2 Fig Cytokine levels were measured by flow cytometry. Cytokine levels were measured by flow cytometry. For the selected cytokines, we did not observe significant difference (KC: p-value = 0.663; IL1: p-vaue = 0.66; IL4: p-value = 0.66; IL5: p-value = 0.663; IL6: p-value = 0.663; IL17: p-value = 0.661) between AuNPs and CNTs exposed and control groups. Data are mean ±SD.
(TIF)

S3 Fig Oxidative stress in mice lung samples in response to AuNP and CNT exposure. Oxidative stress in mice lung samples in response to AuNP and CNT exposure. GSSG/GSH ratio (Wilcoxon test; *p* = 0.173) was measured in mouse lung samples to quantify the level of oxidative stress. GSSG: oxidized form of glutathione disulfide; GSH: reduced glutathione. Box plot describes the median (line across the box), interquartile range and maximum and minimum values (whiskers). Outliers are shown as colored circles (panel a) beyond the ends of whiskers.
(TIF)

S4 Fig DNA damage profile of AuNPs and CNTs in exposed and control mice. DNA damage profile of AuNPs and CNTs in exposed and control mice. DNA damage was assessed by Comet assay. Comet tail is a marker of DNA damage and was not significant (Wilcoxon test; *p* = 0.486) between exposed and control samples. Box plot describes the median (line across the box), interquartile range and maximum and minimum values (whiskers). Outliers are colored circles beyond the ends of whiskers.
(TIF)

S1 Table List of genes investigated for their promoter methylation by bisulfite-PCR pyrosequencing. (DOCX)

S2 Table Genomic locations of CpGs investigated for their promoter methylation by bisulfite-PCR pyrosequencing. (DOCX)

S3 Table Gene-specific methylation assay sequences for bisulfite-PCR pyrosequencing. (DOCX)

S4 Table Sequences for pyrosequencing control run. (DOCX)

S5 Table Physicochemical characteristic of gold nanoparticles (AuNPs) used in this study with H_2_O and serum treatment. (DOCX)

S6 Table Size distribution of carbon nanotubes (CNTs) used in this study. (DOCX)

S7 Table Effect on gene promoter methylation changes in mouse lung DNA induced by exposure to gold nanoparticles (AuNPs) (S7-a) and CNTs (S7-b). Table shows *P*-values from Wilcoxon testing of average methylation per gene and methylation per CpG within each gene. Number of CpGs analysed are variable (e.g., 10 CpGs were analysed in the promoter region of *Atm* and 6 in the promoter region of *Cdk*). Cells in red indicate significant effects of exposure on gene promoter methylation, and cells in orange indicate the effect of exposure close to pre-set cut-off value of significance (borderline significant effect) (Wilcoxon test, *p*-value 0.05 set as significant), *p*-values computed by Mann-Whitney U statistics.
(DOCX)

S8 Table Effect on gene promoter methylation changes in mouse blood DNA induced by exposure to AuNPs (S8 Table a) and SWCNTs and MWCNTs (S8 Table b). Table shows *P*-values of Wilcoxon testing of average methylation per gene and methylation per CpG within each gene. Number of CpGs analysed are variable (e.g., 8 CpGs were analysed in the promoter region of *Atm* and 4 CpGs in the promoter region of *Cdk*). Cells in red indicate the significant effects of exposure on gene promoter methylation, and cells highlighted in orange indicate the effect of exposure close to pre-set cut-off value of significance (borderline significant effect) (Wilcoxon test, *p*-value 0.05 set as significant), *p*-values computed by Mann-Whitney U statistics. DNA coordinates of CpGs analysed in each bisulfite-PCR pyrosequencing promoter assay were the same between lung and blood DNA samples exposed to AuNPs and CNTs (S7a and S7b Table and S8 Table a-b). However, CpGs in blood DNA (S8 Table a-b) that did not pass the quality control were not included in the analysis (e.g., *Atm*: CpG#9, CpG#10 were discarded).
(DOCX)
