# Supplementary figures and images for: Changes in DNA Methylation in Mouse Lungs after a Single Intra-Tracheal Administration of Nanomaterials
Source: PLoS One. 2017 Jan 12;12(1):e0169886. doi: 10.1371/journal.pone.0169886 (PMC5231360; doi:10.1371/journal.pone.0169886)

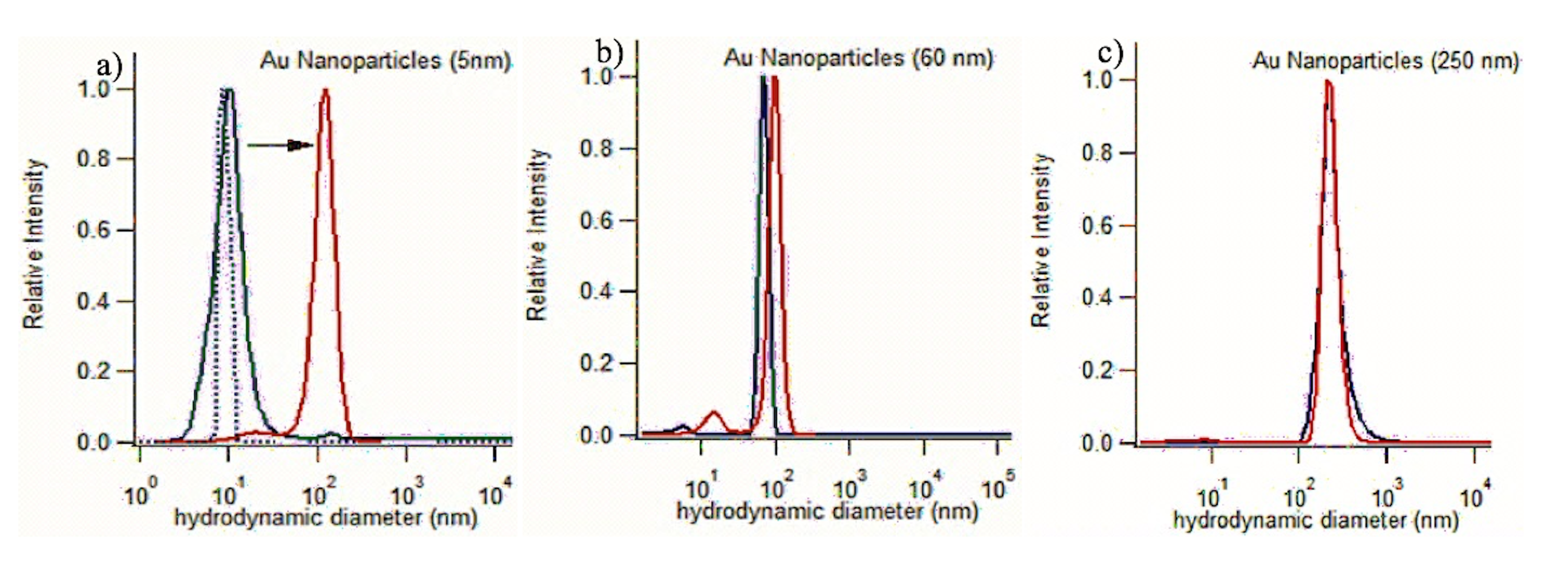

Supplement: S1 Fig — (TIF) [file pone.0169886.s001.tif]

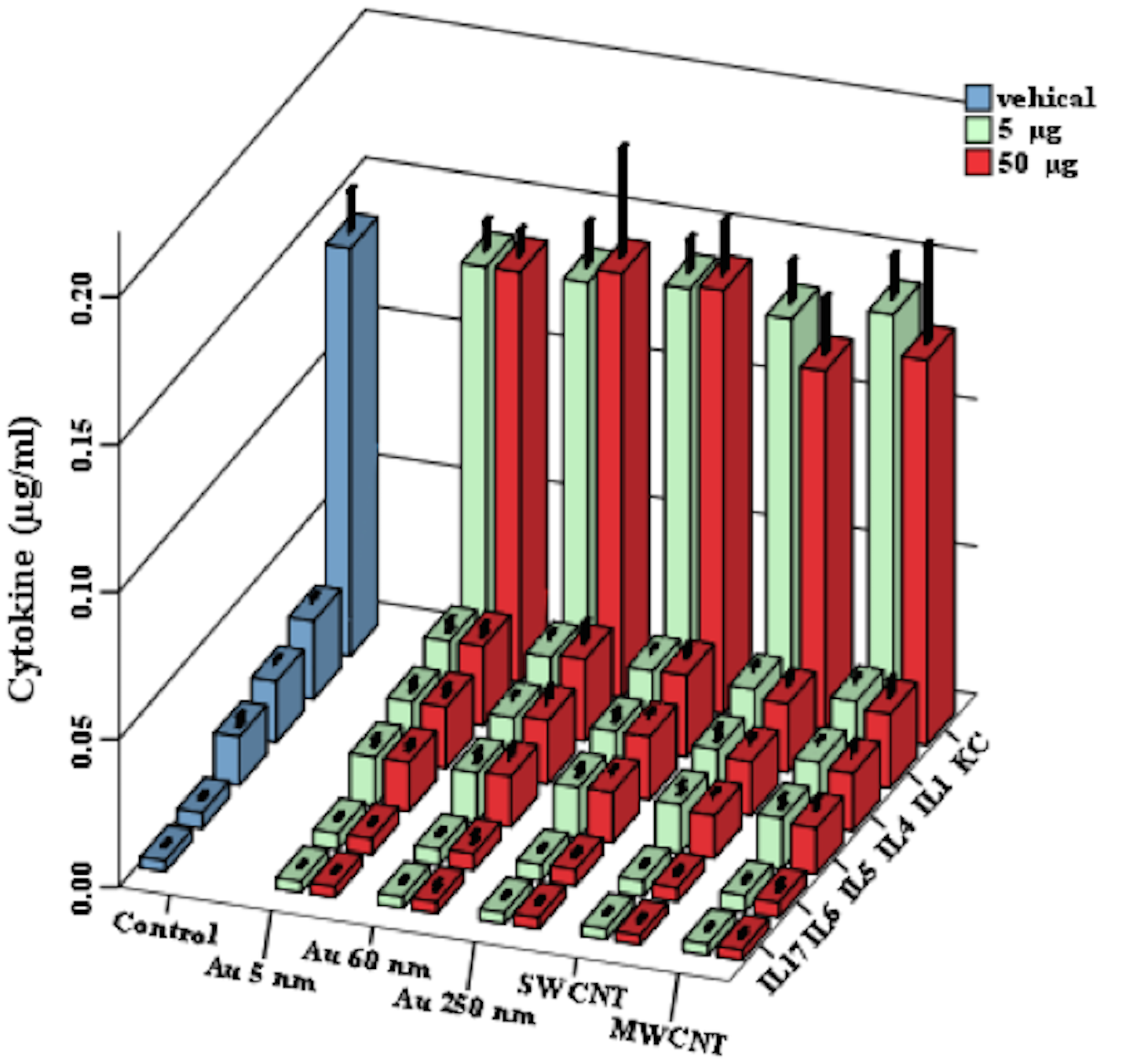

Supplement: S2 Fig — Cytokine levels were measured by flow cytometry. For the selected cytokines, we did not observe significant difference (KC: p-value = 0.663; IL1: p-vaue = 0.66; IL4: p-value = 0.66; IL5: p-value = 0.663; IL6: p-value = 0.663; IL17: p-value = 0.661) between AuNPs and CNTs exposed and control groups. Data are mean ±SD. (TIF) [file pone.0169886.s002.tif]

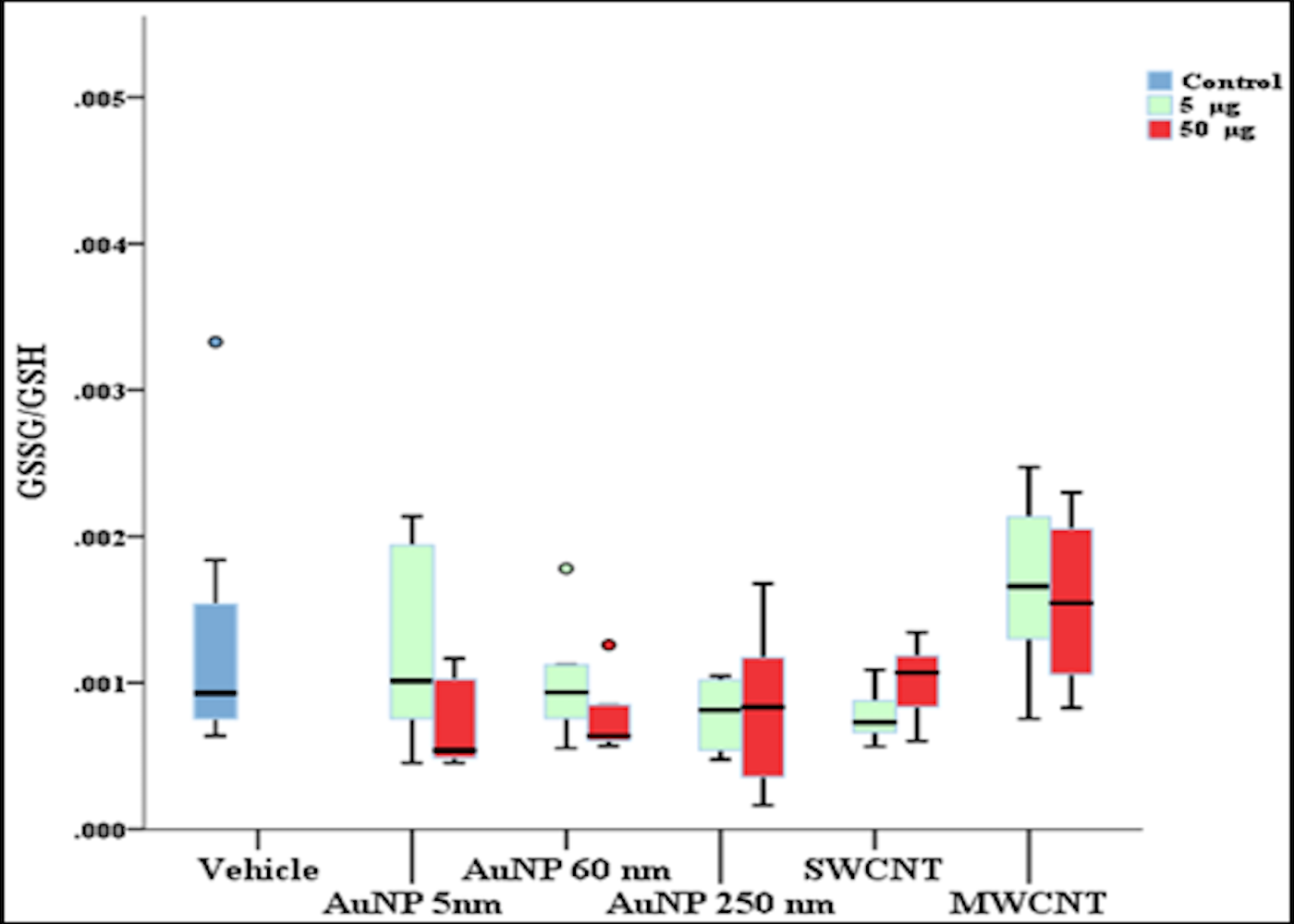

Supplement: S3 Fig — Oxidative stress in mice lung samples in response to AuNP and CNT exposure. GSSG/GSH ratio (Wilcoxon test; p = 0.173) was measured in mouse lung samples to quantify the level of oxidative stress. GSSG: oxidized form of glutathione disulfide; GSH: reduced glutathione. Box plot describes the median (line across the box), interquartile range and maximum and minimum values (whiskers). Outliers are shown as colored circles (panel a) beyond the ends of whiskers. (TIF) [file pone.0169886.s003.tif]

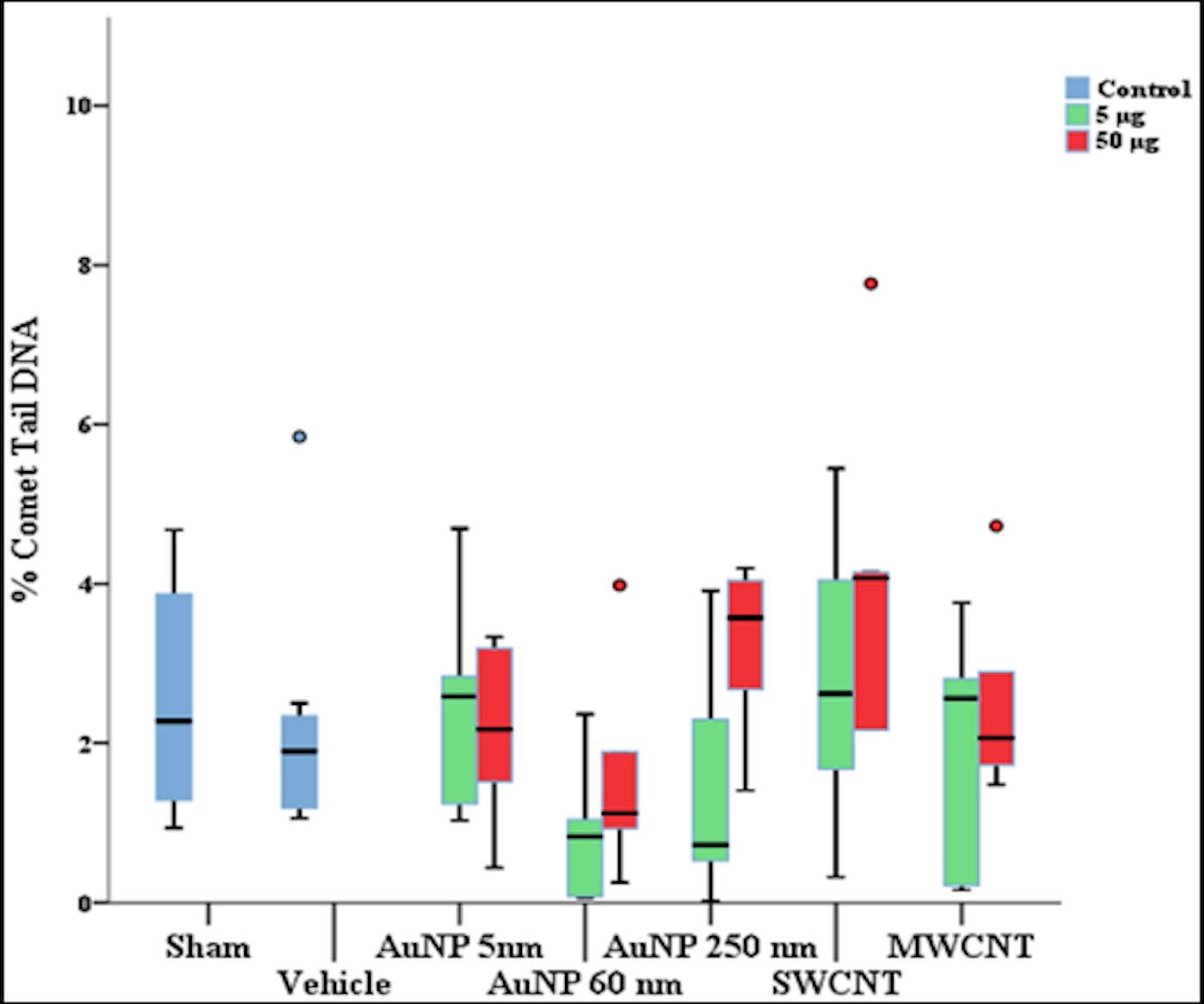

Supplement: S4 Fig — DNA damage profile of AuNPs and CNTs in exposed and control mice. DNA damage was assessed by Comet assay. Comet tail is a marker of DNA damage and was not significant (Wilcoxon test; p = 0.486) between exposed and control samples. Box plot describes the median (line across the box), interquartile range and maximum and minimum values (whiskers). Outliers are colored circles beyond the ends of whiskers. (TIF) [file pone.0169886.s004.tif]
