# Supplementary material for: Changes in DNA Methylation in Mouse Lungs after a Single Intra-Tracheal Administration of Nanomaterials
Source: PLoS One. 2017 Jan 12;12(1):e0169886. doi: 10.1371/journal.pone.0169886 (PMC5231360; doi:10.1371/journal.pone.0169886)
Supplement: S1 Table — (DOCX) [file pone.0169886.s005.docx]

**S1 Table:**

| **Gene symbol** | **NCBI gene ID** | **Chr no** | **Gene name** |
| --- | --- | --- | --- |
| *Atm* | 11920 | 9 | Ataxia telangiectasia mutated |
| *Cdk* | 12566 | 10 | Cyclin-dependent kinase |
| *Dnmt1* | 13433 | 9 | DNA (cytosine-5)-methyltransferase 1 |
| *Gad45a* | 13197 | 6 | Growth arrest and DNA-damage-inducible 45 alpha |
| *Gpx* | 14775 | 9 | Glutathione peroxidase |
| *Gsr* | 14782 | 8 | Glutathione reductase |
| *Gss* | 14854 | 2 | Glutathione synthetase |
| *Myc* | 17869 | 15 | Myelocytomatosis oncogene |
| *Nfkb1* | 18033 | 3 | Nfkb1 nuclear factor of kappa light polypeptide gene enhancer in B cells 1, p105 |
| *Nfkb2* | 18034 | 19 | Nuclear factor of kappa light polypeptide gene enhancer in B cells 2, p49/p100 |
| *Oxsr1* | 108737 | 9 | Oxidative-stress responsive 1 |
| *Tdg* | 21665 | 10 | Thymine DNA glycosylase |
| *Trp53* | 22059 | 11 | Tumor suppressor protein p53 |
| *Trp73* | 22062 | 4 | Tumor suppressor protein p73 |
| *Pparg* | 19016 | 6 | Peroxisome proliferator activated receptor gamma |
| *Tet1* | 52463 | 10 | Tet methylcytosine dioxygenase 1 |
| *Tet2* | 214133 | 3 | Tet methylcytosine dioxygenase 2 |
| *Tnf-a* | 21926 | 17 | Tumor necrosis factor alpha |
| *Xrcc1* | 22594 | 7 | X-ray repair complementing defective repair in Chinese hamster cells 1 |

Chr no: chromosome numbe
