# Supplementary material for: Changes in DNA Methylation in Mouse Lungs after a Single Intra-Tracheal Administration of Nanomaterials
Source: PLoS One. 2017 Jan 12;12(1):e0169886. doi: 10.1371/journal.pone.0169886 (PMC5231360; doi:10.1371/journal.pone.0169886)
Supplement: S2 Table — (DOCX) [file pone.0169886.s006.docx]

**S2 Table:**

| **Gene symbol** | **Genomic location of selected CpG** | | | | | | | | | |
| --- | --- | --- | --- | --- | --- | --- | --- | --- | --- | --- |
|  | CpG#1 | CpG#2 | CpG#3 | CpG#4 | CpG#5 | CpG#6 | CpG#7 | CpG#8 | CpG#9 | CpG#10 |
| *Atm* | 53344818 | 53344825 | 53344827 | 53344830 | 53344834 | 53344840 | 53344846 | 53344848 | 53344859 | 53344861 |
| *Cdk* | 128141888 | 128141899 | 128141901 | 128141912 | 128141918 | 128141925 |  |  |  |  |
| *Dnmt1* | 20757265 | 20757273 | 20757282 | 20757284 | 20757286 | 20757288 |  |  |  |  |
| *Gad45a* | 66986696 | 66986705 | 66986707 | 66986710 | 66986716 |  |  |  |  |  |
| *Gpx* | 108241656 | 108241664 | 108241666 | 108241679 | 108241692 | 108241707 | 108241709 |  |  |  |
| *Gsr* | 34764251 | 34764269 | 34764274 | 34764276 | 34764279 | 34764281 | 34764285 |  |  |  |
| *Gss* | 155418399 | 155418406 | 155418409 | 155418412 | 155418414 | 155418431 |  |  |  |  |
| *Myc* | 61817279 | 61817283 | 61817292 | 61817295 | 61817298 |  |  |  |  |  |
| *Nfkb2* | 46379057 | 46379059 | 46379073 | 46379084 | 46379093 |  |  |  |  |  |
| *Oxsr1* | 119231970 | 119231976 | 119231987 | 119231992 | 119231995 | 119232000 |  |  |  |  |
| *Trp53* | 69392738 | 69392748 | 69392755 | 69392758 | 69392777 |  |  |  |  |  |
| *Trp73* | 153514596 | 153514607 | 153514613 | 153514617 | 153514630 | 153514638 | 153514650 |  |  |  |
| *Pparg* | 115311323 | 115311331 | 115311343 |  |  |  |  |  |  |  |
| *Tet1* | 62349924 | 62349929 | 62349940 | 62349948 |  |  |  |  |  |  |
| *Tet2* | 133208028 | 133208035 | 133208037 | 133208054 |  |  |  |  |  |  |
| *Tnf-a* | 35339096 | 35339111 | 35339113 |  |  |  |  |  |  |  |
| *Xrcc1* | 25332025 | 25332035 | 25332045 | 25332054 |  |  |  |  |  |  |
