# Supplementary material for: Changes in DNA Methylation in Mouse Lungs after a Single Intra-Tracheal Administration of Nanomaterials
Source: PLoS One. 2017 Jan 12;12(1):e0169886. doi: 10.1371/journal.pone.0169886 (PMC5231360; doi:10.1371/journal.pone.0169886)
Supplement: S3 Table — (DOCX) [file pone.0169886.s007.docx]

**S3 Table**:

| **Gene symbol** | **Forward primer** | **Reverse primer (biotinylated)** | **Sequencing primer** | **Annealing temperature (^o^C)** |
| --- | --- | --- | --- | --- |
| ***Atm*** | GGGTGTTTTAAAGGAAGAAGT | TATAACCAAAAAAAACCTAATAACC | TTTAAAGGAAGAAGT | 52 |
| ***Cdk*** | TTGTTTTTGGTTTTGTATATTATTT | TTATCAAAAACTAAACTCTCCTTAC | AAGTTTTTTATGGAG | 52 |
| ***Dnmt1*** | GTTGGTATTTTGTAGGTTGTAGA | ACATAATCTTCCCCCACTCTCTTA | TAGAATAGTTTTGAA | 58.3 |
| ***Gadd45a*** | TTTAGTAGATTTTGGGTTGTAGTTT | TAACTCTACAAATCCATTTCACCCT | TATATAGTGTAGGTT | 53.9 |
| ***Gpx1*** | TTAAAAGGAGGTGTAGGGTTTTGT | CAAAAAACCCAAACTCACAAACT | AGTATGTGTGTTGTT | 58.3 |
| ***Gsr*** | GGAGAGTTATAAGTTGGGTGGTATTT | TTTTTAACTTCAACATTACACCTATACAAA | GTTGGGTGGTATTTG | 53.9 |
| ***Gss*** | TTTTTTTTAAGGAAATTTGATTTT | ATCACTACTCATATAATACCCCTTCC | AGGAAATTTGATTTT | 50 |
| ***Myc*** | TTAAGAAGGTAGTTTTGGAGTGAGAG | AACAAAAAACACTATCCCCAAATAA | GTAAGAGTTTTTTTT | 53.9 |
| ***Nfkb2*** | GTAGAATTGGAGTTGGGTGATATTT | TTACTCCTCTCCAACCAAAAAACT | AGTTGGGTGATATTT | 59.4 |
| ***Oxsr1*** | GGGAATTTGATTTTTAGTTTTTAT | AAACCTTTTATCCTAATTAACCTTC | AAAATTTTTTAGGAT | 50.7 |
| ***Trp53*** | GAATTTAAAGTAATTATTAGGGAA | AAAACCCAAAATTCAAACTACAACT | GTGTTTAAAGTTAAG | 50.7 |
| ***Trp73*** | TTGTAATTTAGGGGTTTAGGAGTGTT | AACTATAATCTCTACCAAACAAATC | GAATATTGAAAGTTT | 53.9 |
| ***Pparg*** | GGAGTTTGTGAGATTAATAGTTTGA | ATCACCTAACCAATCAAATCCAA | AGATTAATAGTTTGA | 53.9 |
| ***Tet1*** | ATTAATTTTTGGATAAATTTTTTAG | AATCATATACCTCTACCTACCTCTTCTAC | GGATAAATTTTTTAG | 52 |
| ***Tet2*** | GTTTATTTTTTGTTTATTTTGGTTA | CATTAAAAACTACTAACTTAATTCTTTC | TGTTTATTTTGGTTA | 50.7 |
| ***Tnf-a*** | TTTTTTTGGTGGAGAAAATTATGAT | CTAATTAACCCCAAATTACCACAAA | TTTATATTTTTGTTT | 53.9 |
| ***Xrcc1*** | GGTTTAATGATTAGGGTAAATTATA | AATTCCCTTAACAACAAACATTCC | AGGTTTTTAGGAAGT | 52 |
| ***Nfkb1*** | AGGGGTTTGGGTATATTTTTTTAAA | AAAAAACCCCAAACAAAAATC | GTAAGAGTTTTTTTT* | 53.9 |
| ***Tdg*** | GGTTTTAAGTTTTTTTGAAGGTTT | ATTAACCTAACCAACATCAC | TTTTTTTGAAGGTTT* | 52.1 |

* Sequencing primers did not pass the validation step for these assays.
