# Supplementary material for: Changes in DNA Methylation in Mouse Lungs after a Single Intra-Tracheal Administration of Nanomaterials
Source: PLoS One. 2017 Jan 12;12(1):e0169886. doi: 10.1371/journal.pone.0169886 (PMC5231360; doi:10.1371/journal.pone.0169886)
Supplement: S4 Table — (DOCX) [file pone.0169886.s008.docx]

**S4 Table**:

| **Assay name** | **Assay sequence** |
| --- | --- |
| **PSQ-C** | TTGCGATACGACGGGAACAAACGTTGAATTC |
| **PSQ-T** | TTGCGATACAACGGGAACAAACGTTGAATTC |
| **Sequencing primer** | AACGTTTGTTCCCGT |
