# Supplementary material for: Changes in DNA Methylation in Mouse Lungs after a Single Intra-Tracheal Administration of Nanomaterials
Source: PLoS One. 2017 Jan 12;12(1):e0169886. doi: 10.1371/journal.pone.0169886 (PMC5231360; doi:10.1371/journal.pone.0169886)
Supplement: S5 Table — (DOCX) [file pone.0169886.s009.docx]

**S5 Table**:

| AuNP | Mean diameter  (nm) [TEM]* | H_2_O  [DLS]** | | 2% serum  [DLS]** | |
| --- | --- | --- | --- | --- | --- |
|  |  | Mean hydrodynamic diameter (nm) | Zeta potential | Mean hydrodynamic diameter (nm) | Zeta potential |
| 5 nm | 5 | 9.6 | -3.99 | 121.6 | -18.42 |
| 60 nm | 60 | 69.3 | -11.49 | 98.1 | -0.54 |
| 250 nm | 250 | 226.5 | -21.17 | 225.8 | -7.15 |

*TEM: transmission electron microscopy, ** DLS: dynamic light scattering, Au NP: gold nanoparticles
