# Supplementary material for: Changes in DNA Methylation in Mouse Lungs after a Single Intra-Tracheal Administration of Nanomaterials
Source: PLoS One. 2017 Jan 12;12(1):e0169886. doi: 10.1371/journal.pone.0169886 (PMC5231360; doi:10.1371/journal.pone.0169886)
Supplement: S6 Table — (DOCX) [file pone.0169886.s010.docx]

**S6 Table**:

| **Nanoparticle** | **Mean diameter (nm)** | **Mean length (μm)** |
| --- | --- | --- |
| SWCNT* | 0.8 | 10 |
| MWCNT** | 9.5 | 105 |

*SWCNT: single-walled CNTs, **MWCNT: multi-walled CNTs
