# Supplementary material for: Changes in DNA Methylation in Mouse Lungs after a Single Intra-Tracheal Administration of Nanomaterials
Source: PLoS One. 2017 Jan 12;12(1):e0169886. doi: 10.1371/journal.pone.0169886 (PMC5231360; doi:10.1371/journal.pone.0169886)
Supplement: S7 Table — Table shows P-values from Wilcoxon testing of average methylation per gene and methylation per CpG within each gene. Number of CpGs analysed are variable (e.g., 10 CpGs were analysed in the promoter region of Atm and 6 in the promoter region of Cdk). Cells in red indicate significant effects of exposure on gene promoter methylation, and cells in orange indicate the effect of exposure close to pre-set cut-off value of significance (borderline significant effect) (Wilcoxon test, p-value 0.05 set as significant), p-values computed by Mann-Whitney U statistics. (DOCX) [file pone.0169886.s011.docx]

**S7 Table**.

**S7 Table a**

| **Gene symbol** | ***Comparing exposed to control groups*** | | | | | | | | | |  |
| --- | --- | --- | --- | --- | --- | --- | --- | --- | --- | --- | --- |
|  | **CpG#1** | **CpG#2** | **CpG#3** | **CpG#4** | **CpG#5** | **CpG#6** | **CpG#7** | **CpG#8** | **CpG#9** | **CpG#10** | **average** |
| ***Atm*** | 0.32 | 0.81 | 0.87 | 0.20 | 0.80 | 0.62 | 0.58 | 0.848 | 0.7524 | **0.004** | 0.38 |
| ***Cdk*** | 0.98 | 0.23 | 0.99 | 0.45 | 0.27 | **0.01** |  |  |  |  | 0.12 |
| ***Dnmt1*** | **0.05** | 0.92 | 0.67 | 0.07 | 0.43 | 0.51 |  |  |  |  | 0.08 |
| ***Gad45a*** | 0.62 | 0.19 | 0.47 | 0.14 | 0.43 |  |  |  |  |  | 0.71 |
| ***Gpx*** | 0.51 | 0.68 | **0.05** | 0.56 | 0.09 | 0.69 | 0.23 |  |  |  | 0.27 |
| ***Gsr*** | **0.01** | 0.57 | 0.21 | **0.02** | 0.62 | **0.004** | 0.29 |  |  |  | **0.02** |
| ***Gss*** | 0.49 | 0.81 | 0.85 | 0.38 | 0.05 | 0.09 |  |  |  |  | 0.11 |
| ***Myc*** | 0.71 | 0.06 | 0.08 | 0.53 | **0.02** |  |  |  |  |  | **0.04** |
| ***Nfkb2*** | 0.95 | **0.03** | 0.44 | 0.59 | 0.80 |  |  |  |  |  | 0.60 |
| ***Oxsr1*** | 0.43 | 0.36 | 0.11 | 0.74 | 0.61 | 0.50 |  |  |  |  | 0.67 |
| ***Trp53*** | **0.01** | 0.46 | 0.97 | 0.31 | 0.73 |  |  |  |  |  | 0.19 |
| ***Trp73*** | 0.51 | 0.46 | 0.88 | 0.42 | 0.49 | 0.06 | 0.46 |  |  |  | 0.49 |
| ***Pparg*** | 0.18 | 0.23 | 0.33 |  |  |  |  |  |  |  | 0.14 |
| ***Tet1*** | 0.14 | 0.18 | 0.64 | 0.23 |  |  |  |  |  |  | 0.42 |
| ***Tet2*** | **0.05** | 0.22 | 0.49 | 0.21 |  |  |  |  |  |  | 0.14 |
| ***Tnf-a*** | 0.92 | 0.51 | 0.56 |  |  |  |  |  |  |  | 0.96 |
| ***Xrcc1*** | 0.25 | 0.67 | **0.02** | **0.04** |  |  |  |  |  |  | **0.04** |

**S7 Table b**

| **Gene symbol** | ***Comparing exposed to control groups*** | | | | | | | | | | |
| --- | --- | --- | --- | --- | --- | --- | --- | --- | --- | --- | --- |
|  | **CpG#1** | **CpG#2** | **CpG#3** | **CpG#4** | **CpG#5** | **CpG#6** | **CpG#7** | **CpG#8** | **CpG#9** | **CpG#10** | **average** |
| ***Atm*** | 0.41 | **0.03** | 0.28 | 0.23 | **0.05** | 0.16 | 0.11 | 0.0787 | 0.1521 | 0.2134 | **0.03** |
| ***Cdk*** | 0.47 | 0.20 | 0.99 | 1.00 | 0.92 | 0.27 |  |  |  |  | 0.80 |
| ***Dnmt1*** | 0.49 | 0.44 | 0.62 | 0.14 | 0.29 | 0.55 |  |  |  |  | 0.12 |
| ***Gad45a*** | 0.92 | 0.17 | **0.02** | 0.06 | 0.07 |  |  |  |  |  | 0.02 |
| ***Gpx*** | 0.94 | 0.46 | 0.20 | 0.25 | 0.62 | 0.39 | 0.10 |  |  |  | 0.78 |
| ***Gsr*** | 0.83 | 0.88 | 0.21 | 0.84 | 0.95 | 0.40 | 0.69 |  |  |  | 0.57 |
| ***Gss*** | 0.35 | **0.05** | 0.77 | 0.57 | 0.52 | 0.93 |  |  |  |  | 0.69 |
| ***Myc*** | 0.46 | 0.51 | 0.58 | 0.71 | 0.51 |  |  |  |  |  | 0.79 |
| ***Nfkb2*** | 0.88 | 0.69 | 0.54 | 0.35 | 0.35 |  |  |  |  |  | 0.59 |
| ***Oxsr1*** | 0.49 | 0.38 | 0.85 | 0.60 | 0.47 | 0.37 |  |  |  |  | 0.44 |
| ***Trp53*** | 0.42 | 0.64 | 0.36 | 0.38 | 0.17 |  |  |  |  |  | 0.50 |
| ***Trp73*** | 0.95 | 0.53 | 0.71 | 0.72 | 0.86 | 0.55 | 0.47 |  |  |  | 0.95 |
| ***Pparg*** | 0.55 | 0.17 | 0.85 |  |  |  |  |  |  |  | 0.55 |
| ***Tet1*** | 0.19 | 0.96 | 0.28 | 0.87 |  |  |  |  |  |  | 0.76 |
| ***Tet2*** | 0.84 | 0.15 | 0.75 | 0.53 |  |  |  |  |  |  | 0.31 |
| ***Tnf-a*** | 0.77 | 0.60 | 0.42 |  |  |  |  |  |  |  | 0.72 |
| ***Xrcc1*** | 0.82 | 0.65 | 0.45 | 0.42 |  |  |  |  |  |  | 0.66 |
