# Supplementary material for: Changes in DNA Methylation in Mouse Lungs after a Single Intra-Tracheal Administration of Nanomaterials
Source: PLoS One. 2017 Jan 12;12(1):e0169886. doi: 10.1371/journal.pone.0169886 (PMC5231360; doi:10.1371/journal.pone.0169886)
Supplement: S8 Table — Table shows P-values of Wilcoxon testing of average methylation per gene and methylation per CpG within each gene. Number of CpGs analysed are variable (e.g., 8 CpGs were analysed in the promoter region of Atm and 4 CpGs in the promoter region of Cdk). Cells in red indicate the significant effects of exposure on gene promoter methylation, and cells highlighted in orange indicate the effect of exposure close to pre-set cut-off value of significance (borderline significant effect) (Wilcoxon test, p-value 0.05 set as significant), p-values computed by Mann-Whitney U statistics. DNA coordinates of CpGs analysed in each bisulfite-PCR pyrosequencing promoter assay were the same between lung and blood DNA samples exposed to AuNPs and CNTs (S7a and S7b Table and S8 Table a-b). However, CpGs in blood DNA (S8 Table a-b) that did not pass the quality control were not included in the analysis (e.g., Atm: CpG#9, CpG#10 were discarded). (DOCX) [file pone.0169886.s012.docx]

**S8 Table.**

**S8 Table a**

| **Gene symbol** | ***Comparing exposed to control groups*** | | | | | | | | |
| --- | --- | --- | --- | --- | --- | --- | --- | --- | --- |
|  | **CpG#1** | **CpG#2** | **CpG#3** | **CpG#4** | **CpG#5** | **CpG#6** | **CpG#7** | **CpG#8** | **Average** |
| ***Atm*** | 0.55 | 0.10 | 0.20 | 0.14 | 0.41 | 0.98 | 0.24 | **0.05** | 0.09 |
| ***Cdk*** | 0.65 | 0.85 | 0.22 | 0.72 |  |  |  |  | 0.92 |
| ***Dnmt1*** | 0.67 | 0.77 | 0.41 | 0.43 |  |  |  |  | **0.04** |
| ***Gad45a*** | 0.56 | 0.73 | 0.19 | 0.81 | 0.24 | 0.43 |  |  | 0.12 |
| ***Gpx*** | 0.70 | 0.31 | 0.15 | 0.41 |  |  |  |  | 0.84 |
| ***Gsr*** | 0.02 | 0.32 | 0.97 | 0.59 | 0.69 |  |  |  | 0.75 |
| ***Gss*** | 0.17 | 0.63 | 0.81 | 0.82 | 0.18 |  |  |  | 0.56 |
| ***Myc*** | 0.51 | 0.60 | 0.07 | 0.29 | 0.06 |  |  |  | 0.22 |
| ***Nfkb2*** | 0.76 | 0.86 | 0.78 | **0.02** |  |  |  |  | 0.47 |
| ***Oxsr1*** | 0.64 | **0.04** | 0.29 | 0.08 | 0.23 | 0.26 | 0.22 |  | 0.14 |
| ***Trp53*** | 0.45 | 0.59 | 0.59 | 0.17 | 0.90 |  |  |  | 0.91 |
| ***Trp73*** | 0.95 | 0.43 | 0.33 | 0.68 | 0.67 | 0.23 |  |  | 0.46 |
| ***Pparg*** | 0.27 | 0.46 | **0.02** |  |  |  |  |  | 0.17 |
| ***Tet1*** | 0.12 | 0.64 | 0.23 |  |  |  |  |  | 0.67 |
| ***Tet2*** | 0.60 | 0.36 | 0.76 | 0.82 | 0.06 | 0.15 |  |  | 0.40 |
| ***Tnf-a*** | 0.24 | 0.59 | 0.06 |  |  |  |  |  | 0.33 |
| ***Xrcc1*** | 0.13 | 0.74 | 0.54 | 0.48 |  |  |  |  | 0.29 |

**S8 Table b**

| **Gene symbol** | ***Comparing exposed to control groups*** | | | | | | | | |
| --- | --- | --- | --- | --- | --- | --- | --- | --- | --- |
|  | **CpG#1** | **CpG#2** | **CpG#3** | **CpG#4** | **CpG#5** | **CpG#6** | **CpG#7** | **CpG#8** | **Average** |
| ***Atm*** | 0.51 | 0.32 | 0.78 | 0.35 | 0.22 | 0.07 | 0.36 | 0.36 | 0.34 |
| ***Cdk*** | 0.57 | 0.89 | 0.42 | 0.08 |  |  |  |  | 0.47 |
| ***Dnmt1*** | 0.34 | 0.47 | 0.35 | 0.80 |  |  |  |  | 0.30 |
| ***Gad45a*** | 0.40 | 0.60 | 0.69 | 0.32 | 0.85 | 0.91 |  |  | 0.68 |
| ***Gpx*** | 0.03 | 0.28 | 0.25 | 0.35 |  |  |  |  | 0.57 |
| ***Gsr*** | 0.66 | 0.85 | 0.83 | 0.77 | 0.20 |  |  |  | 0.95 |
| ***Gss*** | 0.41 | 0.55 | 0.76 | 0.92 | 0.11 |  |  |  | 0.55 |
| ***Myc*** | 0.29 | 0.52 | 0.56 | 0.72 | 0.71 |  |  |  | 0.44 |
| ***Nfkb2*** | 0.10 | 0.76 | 0.73 | 0.58 |  |  |  |  | 0.70 |
| ***Oxsr1*** | 0.19 | 0.26 | 0.59 | 0.37 | 0.71 | 0.14 | 0.12 |  | 0.11 |
| ***Trp53*** | 0.81 | 0.27 | 0.78 | 0.34 | 0.79 |  |  |  | 0.39 |
| ***Trp73*** | 0.84 | 0.16 | 0.15 | 0.97 | 0.76 | 0.90 |  |  | 0.55 |
| ***Pparg*** | 0.33 | 0.59 | 0.50 |  |  |  |  |  | 0.93 |
| ***Tet1*** | 0.58 | 0.66 | 0.14 |  |  |  |  |  | 0.93 |
| ***Tet2*** | 0.24 | 0.38 | 0.98 | 0.15 | 0.50 | 0.51 |  |  | 0.41 |
| ***Tnf-a*** | 0.51 | 0.58 | 0.86 |  |  |  |  |  | 0.56 |
| ***Xrcc1*** | 0.59 | 0.62 | 0.65 | 0.64 |  |  |  |  | 0.82 |
